# Novel species of *Huntiella* from naturally-occurring forest trees in Greece and South Africa

**DOI:** 10.3897/mycokeys.69.53205

**Published:** 2020-07-10

**Authors:** FeiFei Liu, Seonju Marincowitz, ShuaiFei Chen, Michael Mbenoun, Panaghiotis Tsopelas, Nikoleta Soulioti, Michael J. Wingfield

**Affiliations:** 1 Department of Biochemistry, Genetics and Microbiology (BGM), Forestry and Agricultural Biotechnology Institute (FABI), University of Pretoria, Pretoria 0028, South Africa University of Pretoria Pretoria South Africa; 2 China Eucalypt Research Centre (CERC), Chinese Academy of Forestry (CAF), ZhanJiang, 524022, GuangDong Province, China Chinese Academy of Forestry GuangDong China; 3 Institute of Mediterranean Forest Ecosystems, Terma Alkmanos, 11528 Athens, Greece Institute of Mediterranean Forest Ecosystems Athens Greece

**Keywords:** Ceratocystidaceae, *Ceratocystis moniliformis* Complex, *Colophospermum mopane*, *
Huntiella
*, *Platanus orientalis*, saprobes, *Senegalia nigrescens*

## Abstract

*Huntiella* species are wood-infecting, filamentous ascomycetes that occur in fresh wounds on a wide variety of tree species. These fungi are mainly known as saprobes although some have been associated with disease symptoms. Six fungal isolates with typical culture characteristics of *Huntiella* spp. were collected from wounds on native forest trees in Greece and South Africa. The aim of this study was to identify these isolates, using morphological characters and multigene phylogenies of the rRNA internal transcribed spacer (ITS) region, portions of the β-tubulin (BT1) and translation elongation factor 1α (TEF-1α) genes. The mating strategies of these fungi were also determined through PCR amplification of mating type genes. The study revealed two new species; one from *Platanus orientalis* in Greece and one from *Colophospermum mopane* and *Senegalia nigrescens* in South Africa. These novel taxa have been provided with the names, *H. hellenica***sp. nov.** and *H. krugeri***sp. nov.**, respectively. The former species was found to have a homothallic and the latter a heterothallic mating system.

## Introduction

*Huntiella* species are members of the family Ceratocystidaceae (Microascales, Sordariomycetes) as defined by De Beer et al. (2014). This family includes 15 genera, namely *Ambrosiella*, *Berkeleyomyces*, *Bretziella*, *Catunica*, *Ceratocystis*, *Chalaropsis*, *Davidsoniella*, *Endoconidiophora*, *Huntiella*, *Meredithiella*, *Phialophoropsis*, *Solaloca*, *Tielaviopsis*, *Toshiolenlla* and *Wolfgangiella* (De Beer et al. 2014, 2017; Mayers et al. 2015, 2020; Nel et al. 2018). The type species of *Huntiella*, *H. moniliformis*, was first isolated from a sweetgum (*Liquidambar styraciflua*) in Texas, USA (Von Schrenk 1903). It was initially described as *Ceratostomella moniliformis* (Hedgcock 1906) and later transferred to *Ceratocystis* (Moreau 1952). When the family Ceratocystidaceae was redefined (De Beer et al. 2014), *Huntiella* was established as a distinct genus, which can be distinguished from *Ceratocystis* and other members of the Ceratocystidaceae, based on their unique morphological features (Davidson 1935; Van Wyk et al. 2006; Wingfield et al. 2013). Most *Huntiella* spp. are easily recognised by a relatively-thick collar plate connecting the ascomatal necks and bases and ascomatal bases that are rough and ornamented with conical spines (Hedgcock 1906). In addition, aleurioconidia are rarely found in *Huntiella* species unlike most species of *Ceratocystissensu stricto* with which they were previously confused (Hedgcock 1906; De Beer et al. 2014; Mbenoun et al. 2016).

Many species in the Ceratocystidaceae are important pathogens of woody plants, including agricultural, fruit and forest tree crops (Kile 1993; Roux and Wingfield 2009). These pathogens result in a multiplicity of symptoms, such as branch and stem cankers, vascular staining, wilt, root rot, die-back and fruit rot (Kile 1993; Harrington 2004; Roux and Wingfield 2009). *Huntiella* spp. are generally considered saprobes or weak pathogens associated with relatively-minor lesions or sap stain of timber (Van Wyk et al. 2004, 2006, 2011; Tarigan et al. 2010; Kamgan Nkuekam et al. 2012; Chen et al. 2013; Mbenoun et al. 2016; Liu et al. 2018). However, there have been a few reports of more severe disease symptoms and even mortality caused by *Huntiella* spp. (Cristobal and Hansen 1962; De Errasti et al. 2015)

*Huntiella* species are most commonly isolated from freshly-made wounds on trees, to which they are vectored by insects, especially sap-feeding beetles in the Nitidulidae (Heath et al. 2009; Kamgan Nkuekam et al. 2012; Mbenoun et al. 2016, 2017). It has been suggested that the relationship between *Huntiella* species and sap beetle is symbiotic and mutually beneficial, as the insects benefit from essential nutritional supplementation from their fungal partners, while the fungi benefit from transportation and access to scanty and ephemeral substrates (Mbenoun et al. unpublished data). Moreover, one species (*H. bhutanensis*) is found in association with the bark beetle *Ips schmutzenhoferi* (Van Wyk et al. 2004), which is similar to various important species of *Endoconidiophora* (De Beer et al. 2014), although the nature of insect-fungus interaction in this association is unknown.

*Huntiella* spp. are particularly interesting in terms of their mating biology. *Huntiella fecunda* and *H. moniliformis* were, for example, shown to exhibit a unisexual mating system, unlike the many heterothallic species found in this genus (Wilson et al. 2015; Liu et al. 2018). More recent studies have revealed a diversity of mating systems in *Huntiella* spp., including those that are homothallic, heterothallic and unisexual (Wilson et al. 2015; Liu et al. 2018). Efforts are consequently being made to collect these fungi, providing a basis for future fungal genetics studies, but also, together with genomics data (Wingfield et al. 2016), to better understand their biology.

*Huntiella* species are most commonly found in tropical and sub-tropical regions of the world (Van Wyk et al. 2004, 2006, 2011; Kamgan Nkuekam et al. 2012; De Errasti et al. 2015; Mbenoun et al. 2014, 2016; Liu et al. 2018). Twenty-nine species are currently recognised in the genus (Liu et al. 2018). These fungi are grouped in three well-supported genealogical lineages that correspond to geographic centres, where they appear to have radiated (Mbenoun et al. 2016; Liu et al. 2018). These include species in an African Clade known only from Africa, an Asian Clade distributed across Asia and an Indo-Pacific Clade found in Australia and Pacific Islands and some parts of Asia. However, the diversity of *Huntiella* in most regions, including especially Europe, North and South America, is largely unexplored.

The objective of this study was to identify two fungal isolates collected from *Platanus orientalis* L. in Greece and four isolates from *Colophospermum mopane* (Benth.) J. Léonard and *Senegalia nigrescens* (Oliv.) P. Hurter in South Africa. These fungi displayed typical culture characteristics of *Huntiella* spp., including rapid growth on agar medium, white fluffy mycelia when young, as well as the production of fruity aroma. Identification was accomplished, based on morphology and multigene phylogenies for the ITS, BT1 and TEF-1α gene regions. Furthermore, we considered the mating biology of these isolates in order to complement our taxonomic studies.

## Materials and methods

### Fungal isolations

Three South African *Huntiella* isolates were collected from fresh wounds of *Colophospermum mopane* in Kruger National Park in April 2009 and another one of the South African isolates was obtained from a broken branch of a *Senegalia nigrescens* tree damaged by elephants in Kruger National Park in June 2010. The isolates from Greece were obtained from the stump of a *Platanus orientalis* tree that was cut about two months before sampling, in a natural forest along the banks of the Spercheios River in Phthiotis Regional Unit during November 2018. Isolation from wood samples was performed using a trapping technique originally described by Grosclaude et al. (1988). This is a standard diagnostic protocol for the isolation of *Ceratocystis platani* (Walter) Engelbrecht & Harrington using freshly-cut twigs of *P. orientalis* as bait (OEPP/EPPO 2014).

Isolates from Greece were made by transferring ascospore masses from the tips of the ascomata on the surface of *Platanus* twig baits, formed on infected wood surface, to 2% malt extract agar (MEA: 20 g Biolab malt extract, 20 g Difco agar, 1 litre water), using a sterile needle under a dissection microscope (Carl Zeiss Co. Ltd., Oberkochen, Germany). The South African isolate was obtained by transferring mycelial strands from infected wood on to MEA. Primary isolations were incubated for 3–7 d at 25 °C. From these isolations, purified cultures from single hyphal tips were prepared for morphological characterisation, phylogenetic analyses and mating-type studies. All purified isolates were deposited in the culture collection (CMW) of the Forestry and Agricultural Biotechnology Institute (FABI), University of Pretoria, South Africa and the living culture collection (PPRI) of the South African National Collection of Fungi (NCF), Roodeplaat, Pretoria, South Africa. The dried-down type specimens were deposited in the National Collection of Fungi (PREM), Roodeplaat, Pretoria, South Africa.

### DNA extraction, PCR and sequencing

All the isolates obtained in this study were used for DNA sequence-based characterisation. Total genomic DNA was extracted from the mycelium of isolates grown on 2% MEA for 3–4 d at 25 °C, using Prepman Ultra Sample Preparation Reagent (Thermo Fisher Scientific, Waltham, MA, USA) following the manufacturer’s protocols. Three gene regions were amplified for sequencing and phylogenetic analyses. These included the Internal Transcribed Spacer (ITS) regions 1 and 2, including the 5.8S rRNA, a partial β-tubulin 1 gene (BT1) and a partial Translation Elongation factor-1α gene (TEF-1α), amplified using the set of primers as described by Liu et al. (2018).

A total volume of 25 μl PCR reaction mixture contained 1 μl of DNA template, 0.5 μl (10 pM) of each primer (Forward and Reverse), 5 μl MyTaq PCR buffer (Bioline GmbH, Germany) and 0.3 μl of MyTaq DNA Polymerase (Bioline GmbH, Germany). The PCR reactions were conducted using an Applied Biosystems ProFlex PCR System (Thermo Fisher Scientific, Waltham, MA, USA). The PCR programme for amplification of the ITS, BT1 and TEF1-α gene regions was as follows: an initial denaturation step at 95 °C for 5 min followed by 35 cycles of 30 s at 95 °C, 45 s at 56 °C and 60 s at 72 °C and a final extension step at 72 °C for 10 min. Amplified fragments were purified using ExoSAP-IT™ PCR Product Cleanup Reagent (Thermo Fisher Scientific, Waltham, MA, USA) to remove excess primers and dNTPs. Amplicons were sequenced in both directions using an ABI PRISM™ 3100 DNA sequencer (Applied Biosystems, USA) at the Sequencing Facility of the Faculty of Natural and Agricultural Sciences, University of Pretoria, Pretoria, South Africa.

### Multi-gene phylogenetic analyses

The programme Geneious v. 7.0 was used to edit and assemble raw sequence reads into contigs (Kearse et al. 2012). Sequence data for representative type isolates of all described *Huntiella* spp. (except *H. decorticans*) were downloaded from GenBank (http://www.ncbi.nlm.nih.gov). The sequences were aligned using MAFFT v. 7 with an online FFT-NS-i strategy (https://mafft.cbrc.jp/alignment/server/; Katoh and Standley 2013) and confirmed visually. Sequences for the novel species discovered in this study were deposited in GenBank.

Single gene sequence datasets of the ITS, BT1 and TEF-1α and the combined dataset of the three gene regions were analysed using Maximum Likelihood (ML), Maximum Parsimony (MP) and Bayesian Inference (BI). The appropriate substitution model for each dataset was obtained using the software package jModeltest v. 2.1.5 (Posada 2008). The ML phylogenetic analyses were conducted using PhyML v. 3.0 (Guindon and Gascuel 2003). Confidence levels for the nodes were determined using 1000 bootstrap replicates. MP analyses were performed using PAUP v. 4.0b10 (Swofford 2003). Gaps were treated as a fifth character. BI analyses were conducted using MrBayes v. 3.2.6 (Ronquist et al. 2012) on the CIPRES Science Gateway v. 3.3. Four Markov Chain Monte Carlo (MCMC) chains were run from a random starting tree for five million generations and trees were sampled every 100 generations. Twenty-five percent of the trees sampled were discarded as burn-in and the remaining trees were used to construct 50% majority rule consensus trees. *Ceratocystis cercfabiensis* (isolate CMW 43029) was used as the outgroup taxon for all the phylogenetic analyses. The resulting trees were visualised using MEGA v. 7.

### Microscopy, growth study and mating-type assignment

Morphological features were studied on the isolates grown on 2% MEA. The fruiting structures were initially mounted in water and this was later replaced with 85% lactic acid and in which measurements were made and images captured. Nikon microscopes (Eclipse N*i*, SMZ 18, Nikon, Tokyo, Japan) mounted with a camera (Nikon DS Ri-2) were used for all observations. Fifty measurements of each relevant microscopic structure were made when available and these are presented as minimum–maximum and average ± standard deviation.

A study of growth in culture was conducted at temperatures from 5–35 °C at 5 °C intervals on the 90 mm Petri dishes containing 2% MEA. A mycelial plug (5 mm diam.) taken from an actively-growing colony was placed at the centres of Petri dishes. Four replicates per isolate were used to study growth rate and the experiment was repeated once. Colony diameters were assessed by taking two measurements perpendicular to each other for all isolates daily and growth rates were calculated. Colony characteristics were described on the same medium used for the growth studies and colours were assessed using the colour charts of Rayner (1970).

The mating type (MAT) of the studied *Huntiella* spp. was determined, based on the results of the mating type PCR reactions (Wilson et al. 2015). Primers, to see which of the MAT genes, Oman_111_F and Oman_111_R were thus used to amplify a 335 bp fragment of the MAT1-1-1 gene and Om_Mo_121_F and Om_Mo_121_R to amplify a 572 bp fragment of the MAT1-2-1 gene, as described by Wilson et al. (2015).

## Results

### Fungal isolations

Six isolates resembling *Huntiella* spp. were included in this study. Two isolates had ascomata with long necks, conical spines on the ascomatal bases and hat-shaped ascospores and four isolates had only thielaviopsis-like asexual state (Van Wyk et al. 1991). Four isolates were collected from *Colophospermum mopane* and *Senegalia nigrescens* in the Kruger National Park of South Africa and two isolates were from *Platanus orientalis* in Greece. Ascomata resembling *Huntiella* spp. were observed on twig baits from *P. orientalis* samples from Greece and two isolates were obtained in pure culture. All isolates obtained in this study have been preserved in the culture collections described above (Table 1).

**Table 1.** List of *Huntiella* species included in this study.

Species^a^	CMW No.^b^	Other No.^b^	GenBank accession No.^c^			Hosts (or substrate)	Origin	Reference
			ITS	BT1	TEF-1α			
*Ceratocystis cercfabiensis*	CMW 43029	CERC 2170;	KP727592	KP727618	KP727643	*Eucalyptus* sp.	China	Liu et al. 2015
		CBS 139654						
*Huntiella ani*	CMW 44684	CERC 2827;	MH118602	MH118635	MH118668	*Eucalyptus* sp.	China	Liu et al. 2018
		CBS 143283						
*H. ani*	CMW 44686	CERC 2829;	MH118603	MH118636	MH118669	*Eucalyptus* sp.	China	Liu et al. 2018
		CBS 143282						
*H. bellula*	CMW 49312	CERC 2854;	MH118607	MH118640	MH118673	*Eucalyptus* sp.	China	Liu et al. 2018
		CBS 143286						
*H. bellula*	CMW 49314	CERC 2862;	MH118610	MH118643	MH118676	*Eucalyptus* sp.	China	Liu et al. 2018
		CBS 143285						
*H. bhutanensis*	CMW 8242	CBS 112907	AY528951	AY528956	AY528961	*Picea spinulosa*	Bhutan	Van Wyk et al. 2004
*H. bhutanensis*	CMW 8217	CBS 114289	AY528957	AY528962	AY528952	*P. spinulosa*	Bhutan	Van Wyk et al. 2004
*H. ceramica*	CMW 15245	CBS 122299	EU245022	EU244994	EU244926	*Eucalyptus grandis*	Malawi	Heath et al. 2009
*H. ceramica*	CMW 15248	CBS 122300	EU245024	EU244996	EU244928	*E. grandis*	Malawi	Heath et al. 2009
*H. chinaeucensis*	CMW 24658	CBS 127185	JQ862729	JQ862717	JQ862741	*Eucalyptus* sp.	China	Chen et al. 2013
*H. chinaeucensis*	CMW 24661	CBS 127186	JQ862731	JQ862719	JQ862743	*Eucalyptus* sp.	China	Chen et al. 2013
*H. chlamydoformis*	CMW 36932	CBS 131674	KF769087	KF769109	KF769098	*Theobroma cacao*	Cameroon	Mbenoun et al. 2016
*H. chlamydoformis*	CMW 37102	CBS 131675	KF769088	KF769110	KF769099	*Terminalia superba*	Cameroon	Mbenoun et al. 2016
*H. confusa*	CMW 43452	CERC 2158;	MH118583	MH118616	MH118649	*Acacia confusa*	China	Liu et al. 2018
		CBS 143577						
*H. confusa*	CMW 43453	CERC 2162;	MH118584	MH118617	MH118650	*A. confusa*	China	Liu et al. 2018
		CBS 143288						
*H. cryptoformis*	CMW 36826	CBS 131277	KC691462	KC691486	KC691510	*Terminalia sericea*	South Africa	Mbenoun et al. 2014
*H. cryptoformis*	CMW 36828	CBS 131279	KC691464	KC691488	KC691512	*Ziziphus mucronata*	South Africa	Mbenoun et al. 2014
*H. decipiens*	CMW 25918	CBS 129735	HQ203218	HQ203235	HQ236437	*E. cloeziana*	South Africa	Kamgan Nkuekam et al. 2013
*H. decipiens*	CMW 25914	CBS 129737	HQ203219	HQ203236	HQ236438	*E. maculata*	South Africa	Kamgan Nkuekam et al. 2013
*H. eucalypti*	CMW 44692	CERC 2840;	MH118605	MH118638	MH118671	*Eucalyptus* sp.	China	Liu et al. 2018
		CBS 143291						
*H. eucalypti*	CMW 44693	CERC 2841;	MH118606	MH118639	MH118672	*Eucalyptus* sp.	China	Liu et al. 2018
		CBS 143290						
*H. fabiensis*	CMW 49307	CERC 2753;	MH118596	MH118629	MH118662	*Eucalyptus* sp.	China	Liu et al. 2018
		CBS 143294						
*H. fabiensis*	CMW 49309	CERC 2763;	MH118599	MH118632	MH118665	*Eucalyptus* sp.	China	Liu et al. 2018
		CBS143292						
*H. fecunda*	CMW 49302	CERC 2449;	MH118586	MH118619	MH118652	*Eucalyptus* sp.	China	Liu et al. 2018
		CBS 143296						
*H. fecunda*	CMW 49303	CERC 2451a;	MH118587	MH118620	MH118653	*Eucalyptus* sp.	China	Liu et al. 2018
		CBS 143295						
*H. glaber*	CMW 43436	CERC 2132;	MH118580	MH118613	MH118646	*E. exserta*	China	Liu et al. 2018
		CBS 143298						
*H. glaber*	CMW 49299	CERC 2133;	MH118581	MH118614	MH118647	*E. exserta*	China	Liu et al. 2018
		CBS 143297						
***H. hellenica***	**CMW 54800**	**PPRI 27982**	**MT524073**	**MT513125**	**MT513131**	***Platanus orientalis***	**Greece**	**Present study**
***H. hellenica***	**CMW 54801**	**PPRI 27983**	**MT524072**	**MT513124**	**MT513130**	***P. orientalis***	**Greece**	**Present study**
*H. inaequabilis*	CMW 44372	CERC 2740;	MH118590	MH118623	MH118656	*Eucalyptus* sp.	China	Liu et al. 2018
		CBS 143300						
*H. inaequabilis*	CMW 49306	CERC 2749;	MH118595	MH118628	MH118661	*Eucalyptus* sp.	China	Liu et al. 2018
		CBS 143299						
*H. inquinana*	CMW 21106		EU588587	EU588666	EU588674	*Acacia mangium*	Indonesia	Tarigan et al. 2010
*H. inquinana*	CMW 21107	CBS 124009	EU588588	EU588667	EU588675	*A. mangium*	Indonesia	Tarigan et al. 2010
***H. krugeri***	**CMW 36849**	**CBS 131676 PPRI 27952**	**MT524068**	**MT513120**	**MT513126**	***A. nigrescens***	**South Africa**	**Present study**
***H. krugeri***	**CMW 55933**		**MT524069**	**MT513121**	**MT513127**	***Colophospermum mopane***	**South Africa**	**Present study**
***H. krugeri***	**CMW 55934**		**MT524070**	**MT513122**	**MT513128**	***C. mopane***	**South Africa**	**Present study**
***H. krugeri***	**CMW 55935**		**MT524071**	**MT513123**	**MT513129**	***C. mopane***	**South Africa**	**Present study**
*H. meiensis*	CMW 44374	CERC 2742;	MH118591	MH118624	MH118657	*Eucalyptus* sp.	China	Liu et al. 2018
		CBS 143302						
*H. meiensis*	CMW 44376	CERC 2746;	MH118594	MH118627	MH118660	*Eucalyptus* sp.	China	Liu et al. 2018
		CBS 143301						
*H. microbasis*	CMW 21117	CBS 124013	EU588593	EU588672	EU588680	*A. mangium*	Indonesia	Tarigan et al. 2010
*H. microbasis*	CMW 21115	CBS 124015	EU588592	EU588671	EU588679	*A. mangium*	Indonesia	Tarigan et al. 2010
*H. moniliformis*	CMW 9590	CBS 116452	AY431101	AY528985	AY529006	*E. grandis*	South Africa	Van Wyk et al. 2006
*H. moniliformis*	CMW 4114	CBS 118151	AY528997	AY528986	AY529007	*Shizolobium parahyba*	Ecuador	Van Wyk et al. 2006
*H. moniliformopsis*	CMW 9986	CBS 109441	AY528998	AY528987	AY529008	*E. obliqua*	Australia	Yuan & Mohammed 2002
*H. moniliformopsis*	CMW 10214	CBS 115792	AY528999	AY528988	AY529009	*E. sieberi*	Australia	Yuan & Mohammed 2002
*H. oblonga*	CMW 23803	CBS 122291	EU245019	EU244991	EU244951	*A. mearnsii*	South Africa	Heath et al. 2009
*H. oblonga*	CMW 23802		EU245020	EU244992	EU244952	*A. mearnsii*	South Africa	Heath et al. 2009
*H. omanensis*	CMW 11048	CBS 115787	DQ074742	DQ074732	DQ074737	*Mangifera indica*	Oman	Al-subhi et al. 2006
*H. omanensis*	CMW 3800	CBS 117839	DQ074743	DQ074733	DQ074738	*M. indica*	Oman	Al-subhi et al. 2006
*H. pycnanthi*	CMW 36916	CBS 131672	KF769096	KF769118	KF769107	*The. cacao*	Cameroon	Mbenoun et al. 2016
*H. pycnanthi*	CMW 36910		KF769095	KF769117	KF769106	*The. cacao*	Cameroon	Mbenoun et al. 2016
*H. salinaria*	CMW 25911	CBS 129733	HQ203213	HQ203230	HQ236432	*E. maculata*	South Africa	Kamgan Nkuekam et al. 2013
*H. salinaria*	CMW 30703	CBS 129734	HQ203214	HQ203231	HQ236433	*E. saligna*	South Africa	Kamgan Nkuekam et al. 2013
*H. savannae*	CMW 17300	CBS 121151	EF408551	EF408565	EF408572	*A. nigrescens*	South Africa	Kamgan Nkuekam et al. 2008
*H. savannae*	CMW 17297		EF408552	EF408566	EF408573	*Combretum zeyheri*	South Africa	Kamgan Nkuekam et al. 2008
*H. sublaevis*	CMW 22449	CBS 122517	FJ151431	FJ151465	FJ151487	*Terminalia ivorensis*	Ecuador	Van Wyk et al. 2011
*H. sublaevis*	CMW 22444	CBS 122518	FJ151430	FJ151464	FJ151486	*T. ivorensis*	Ecuador	Van Wyk et al. 2011
*H. sumatrana*	CMW 21109	CBS 124011	EU588589	EU588668	EU588676	*A. mangium*	Indonesia	Tarigan et al. 2010
*H. sumatrana*	CMW 21111	CBS 124012	EU588590	EU588669	EU588677	*A. mangium*	Indonesia	Tarigan et al. 2010
*H. tribiliformis*	CMW 13011	CBS 115867	AY528991	AY529001	AY529012	*Pinus merkusii*	Indonesia	Van Wyk et al. 2006
*H. tribiliformis*	CMW 13012	CBS 118242	AY528992	AY529002	AY529013	*P. merkusii*	Indonesia	Van Wyk et al. 2006
*H. tyalla*	CMW 28917		HM071899	HM071909	HQ236448	*E. grandis*	Australia	Kamgan Nkuekam et al. 2012
*H. tyalla*	CMW 28920		HM071896	HM071910	HQ236449	*E. grandis*	Australia	Kamgan Nkuekam et al. 2012

^a^ Species indicated in bold are newly described in this study. ^b^ CBS = Westerdijk Fungal Biodiversity Institute, Utrecht, the Netherlands; CERC = Culture collection of China Eucalypt Research Centre (CERC), Chinese Academy of Forestry (CAF), ZhanJiang, GuangDong Province, China; CMW = Culture collection of the Forestry and Agricultural Biotechnology Institute (FABI), University of Pretoria, Pretoria, South Africa; PPRI = The living culture collection (PPRI) of the South African National Collection of Fungi (NCF), Roodeplaat, Pretoria, South Africa. ^c^ GenBank accession numbers indicated in bold are generated in this study.

### Multi-gene phylogenetic analyses

All six isolates, included in this study, were successfully sequenced at all three selected gene regions for phylogenetic analyses, resulting in DNA sequence data of approximately 614, 574 and 830 bp for the ITS, BT1 and TEF-1α gene regions, respectively. These newly-generated sequences were deposited in GenBank (Table 1). Comparisons with reference sequences of previously-described *Huntiella* spp. produced a concatenated sequence alignment which was deposited in TreeBASE (no. 26341).

**Figure 1.**
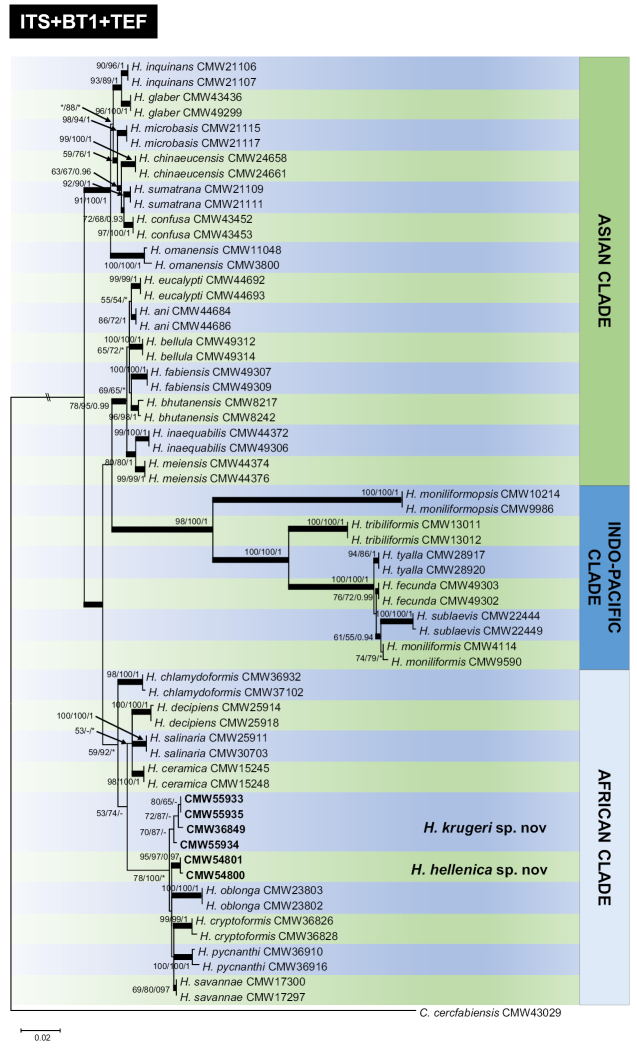
ML tree of *Huntiella* species generated from the combined DNA sequence data of ITS, BT1 and TEF-1α DNA. Sequences generated from this study are printed in bold type. Bold branches indicate posterior probabilities values ≥ 0.9. Bootstrap values and posterior probabilities values are presented above branches as ML/MP/BI. Bootstrap value < 50% or probabilities values < 0.9 are marked with *. Nodes lacking the support value are marked with -. *Ceratocystis cercfabiensis* (CMW 43029) represents the outgroup.

The three tree topologies resulting from ML, MP and BI were concordant and showed similar phylogenetic relationships amongst taxa (Fig. 1, Suppl. materials 1–3: Figs S1–S3). Based on the phylogenetic analyses of the BT1 (Suppl. materil 2, Fig. S2), TEF-1α (Suppl. material 3: Fig. S3) and the combined gene regions (Fig. 1), the six isolates clustered in two well-supported clades, clearly separated from each other and from previously described *Huntiella* spp. The ITS tree (Suppl. material 1: Fig. S1) provided a poor resolution to separate the species. All the isolates grouped in the African Clade of *Huntiella* spp. (Fig. 1).

### Taxonomy

#### Huntiella hellenica

Taxon classificationFungiMicroascalesCeratocystidaceae

F.F. Liu. Marinc. & M.J. Wingf. sp. nov.

C117DE8D-EF83-54C6-A90E-CB0566BEA5F2

MycoBank No: 835637

Fig. 2

##### Etymology.

The name refers to the country, Greece where this fungus was collected.

##### Mating strategy.

Homothallic, with sexually complementary isolates having both the MAT1-1-1 and MAT1-2-1 genes.

***Sexual state*.***Ascomata* produced in 2% MEA in a week, perithecial; *ascomatal bases* mostly embedded in thick or loose mycelial mat, globose to ellipsoidal or obpyriform, pale brown when young, becoming dark brown with age, 173–377 µm long (avg. 238.8 µm), 157–493 µm wide (avg. 218.2 µm), ornamented with spine-like structures, dark brown, conical, 12–29 µm long, 4–9 µm wide at base becoming attenuated; *ostiolar necks* upright, straight, occasionally situated at off-centre of base, darker than base when young, 344–616 µm long (avg. 515.5 µm), 34–60 µm wide (avg. 46.6 µm) at base, gradually tapering towards apex; *ostiolar hyphae* hyaline, straight to divergent, 15–39 µm long, 1–3 µm wide, tapering towards apex. *Asci* evanescent. *Ascospores* hyaline, subglobose, aseptate, covered with sheath giving a hat-like feature in side view, 4–5.5 × 3–4.5 µm (5 ± 0.23 × 4 ± 0.28 µm) excluding sheath.

***Asexual state*.**Thielaviopsis-like *Conidiophores* macronematous, simple or branched; when branched radiating from basal cell once, often reduced to conidiogenous cells. *Conidiogenus cells* endoblastic, hyaline, varying from lageniform to cylindrical depending spore shape; in case of thick barrel-shaped conidia, apex often becoming wider than base. *Conidia* hyaline, 1-celled, in two recognisable shapes; majority ellipsoidal to barrel-shaped (side swollen, ends round), typical fat barrel-shaped 5–8 × 4.5–7.5 µm (5.9 ± 0.61 × 5.3 ± 0.55 µm), width of some barrel-shaped ranging 2.5–4 µm wide; rectangular-shaped (side straight, ends truncated), not commonly found, 5–9 × 1–3 µm (6.9 ± 1.18 × 2.3 ± 0.38 µm). *Aleurioconidia* not observed.

##### Culture characteristics.

Cultures on 2% MEA in dark in 8 d showing circular growth with even edge, mycelium flat, superficial, medium dense and texture becoming pelt-like with age, colour above not uniform, salmon (11f’) to ochreous (15b’) with inner half irregularly umber (13m), below ochreous (15b’) with inner half irregularly umber (13i’) at centre. Optimum growth temperatures at 30 °C at 9.6 mm/d, followed by at 25 °C (7.6 mm/d), 35 °C (7.2 mm/d), 20 °C (4.7 mm/d), 15 °C (3.2 mm/d), 10 °C (1.1 mm/d) and 5 °C (0.2 mm/d).

##### Specimens examined.

Greece, Phthiotis, near the village Kastri, occurring on freshly-cut stumps of *Platanus orientalis* in a natural forest along the banks of the Spercheios River, Nov. 2018, P. Tsopelas & N. Soulioti, PREM 62889, holotype (dried culture of CMW 54800), culture ex-holotype CMW 54800 = PPRI 27982, other cultures CMW 54801 = PPRI 27983.

**Figure 2.**
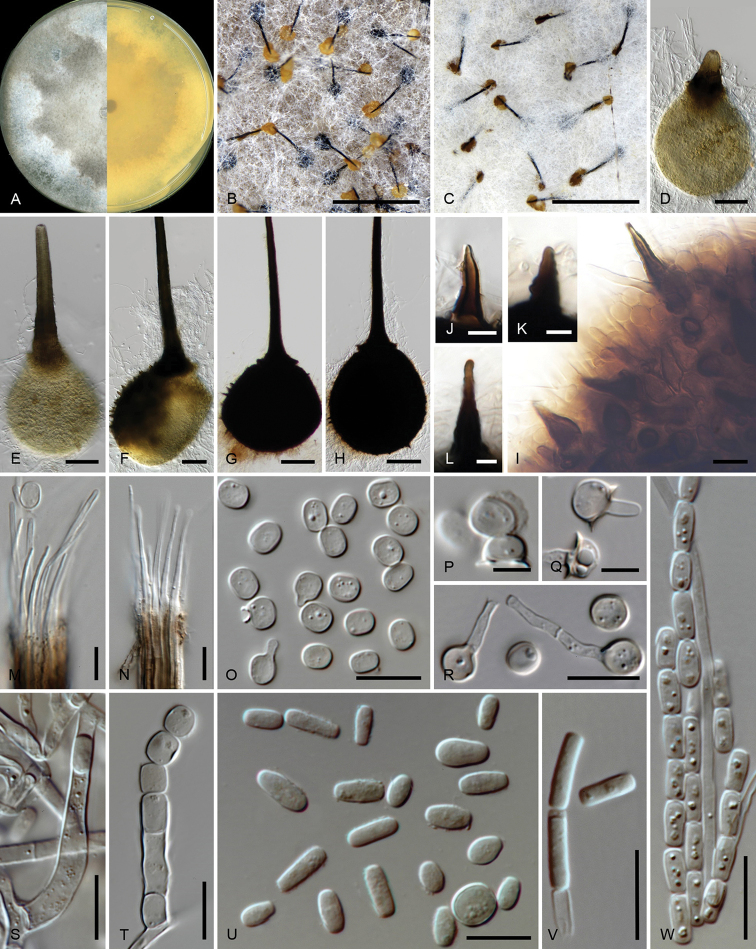
Micrographs of *Huntiella hellenica* sp. nov. (ex-holotype CMW 54800 = PPRI 27982) **A** culture grown on 2% MEA at 30 °C (optimum growth temperature) in the dark for 34 d **B, C** colony with ascomatal base embedded in mycelia with ascospore mass at the tip of ostiolar neck **D–F** young ascoma showing development of ostiolar neck and less-pigmented base **G, H** mature ascoma ornamented with spines **I** close-up of ascomatal wall showing spines **J–L** close up of ornament (spin-like) **M, N** Ostiolar hyphae **O** Ascospores **P** Ascospores covered with sheath appearing like a hat **Q, R** Germinating ascospores **S** Lageniform conidiogenous cell **T** Cylindrical-shape conidiogenous cell **U** Conidia in various shapes from diverse barrel-shaped to rectangular-shaped **V** rectangular-shaped conidia **W** chains of conidia. Scale bars: 1 mm (**B, C**); 50 µm (**D–H**); 10 µm (**I–W**).

##### Notes.

The sexual state of *H. hellenica* developed at temperatures over 25 °C. Cultures incubated at 20 °C and below produced only the asexual state. *Huntiella hellenica* is closely related to *H. savannae* (Kamgan Nkuekam et al. 2008), *H. pycnanthi* (Mbenoun et al. 2016) and *H. krugeri*. It can, however, be distinguished from these two species by the dimensions of ascomatal necks and barrel-shaped conidia and growth rate. *Huntiella hellenica* produced shorter (average 515.5 μm long) ascomatal necks than *H. savannae* (average 579 μm long) and *H. pycnanthi* (average 673 μm long). *Huntiella hellenica* had larger (average 6.9 × 2.3 μm) barrel-shaped conidia than *H. savannae* (average 4.8 × 3 μm) and *H. pycnanthi* (average 6 × 3 μm). Optimal temperature for growth of *H. hellenica* was 30 °C, similar to *H. savannae* and *H. pycnanthi*, but *H. hellenica* differed from *H. pycnanthi* in growing minimally at 10 °C and below.

#### Huntiella krugeri

Taxon classificationFungiMicroascalesCeratocystidaceae

F.F. Liu. Marinc. & M.J. Wingf. sp. nov.

60B4330B-1F8B-575A-AAA0-A8F5AE420CAC

MycoBank No: 835638

Fig. 3

##### Etymology.

The name refers to the Kruger National Park in South Africa, where this fungus was collected.

##### Mating strategy.

Heterothallic with isolates having either a MAT1-1-1 gene or a MAT1-2-1 gene.

***Sexual state*.** Not observed.

***Asexual state***. Produced on 2% MEA in 3 weeks. Thielaviopsis-like. *Conidiophores* macronematous, upright, simple or branched in one tier, 29–37 µm in length, often reduced to conidiogenous cells; *Conidiogenous cells* enteroblastic, lageniform, 10–20 µm long, 1.5–3 µm wide, tapering towards apex. *Conidia* hyaline, rectangular-shaped, usually straight, with top-end conidium often club-shaped, 4–11 × 1–2 µm (avg. 6.2 × 1.7 µm). *Aleurioconidia* hyaline, holoblastic, mostly terminal, ellipsoidal to subglobose with an extended tube-like base, club-shaped, 4–7 × 2–3 µm (5.6 ± 0.76 × 2.5 ± 0.24 µm).

**Figure 3.**
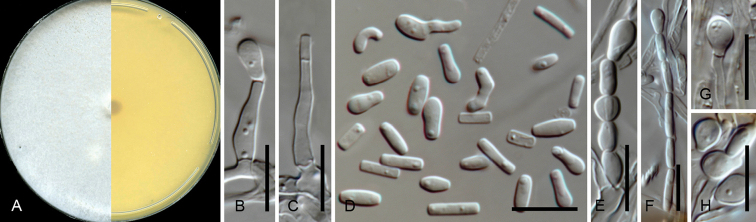
Micrographs of *Huntiella krugeri* sp. nov. (ex-holotype CMW 36849 = CBS 131676 = PPRI 27952). **A** Culture grown on 2% MEA in the dark for 34 d **B, C** Conidiogenous cell **D** Conidia in various shapes **E** Chain of conidia in different shapes **F** Chain of rectangular-shaped conidia with top-end of club-shaped **G, H**Aleurioconidia. Scale bars: 10 µm (**B–H**).

##### Culture characteristics.

Cultures on 2% MEA in dark in 8 d showing circular growth with even edge, mycelium superficial, flat, dense, colour above uniformly white, below luteous (19). Optimum growth temperatures were at 30 °C at 9 mm/d, followed by at 25 °C (8.2 mm/d), 35 °C (6.2 mm/d), 20 °C (6 mm/d), 15 °C (3.4 mm/d), 10 °C (0.9 mm/d) and 5 °C (0.3 mm/d).

##### Specimens examined.

South Africa, Mpumalanga, Kruger National Park, Satara rest camp, *Senegalia nigrescens*, June 2010, M. Mbenoun, PREM 62883, holotype (dried culture of CMW 36849), culture ex-holotype CMW 36849 = CBS 131676 = PPRI 27952.

##### Other cultures.

South Africa, Mpumalanga, Kruger National Park, Punda Maria, *Colophospermum mopane*, April 2009, M. Mbenoun, CMW 55933, CMW 55934, CMW 55935.

##### Notes.

*Huntiella krugeri* is closely related to *H. hellenica* described in the present study, *H. cryptoformis* (Mbenoun et al. 2014) and *H. savannae* (Kamgan Nkuekam et al. 2008). Due to its heterothallic nature, *H. krugeri* produced only the asexual state in this study. The bacilliform conidia of *H. krugeri* (average 6.2 × 1.7 μm) were longer than those of *H. hellenica* (average 5.9 × 5.3 μm) and *H. cryptoformis* (average 5.5 × 2.5 μm). In addition, *H. krugeri* produced hyaline aleurioconidia, which are absent in other closely-related species in the genus.

## Discussion

This study led to the discovery of two novel *Huntiella* species isolated from *Platanus orientalis* in Greece, *Colophospermum mopane* and *Senegalia nigrescens* in the Kruger National Park of South Africa. These two species, provided with the name *H. hellenica* and *H. krugeri*, respectively, were shown to reside in the African Clade of *Huntiella* (Mbenoun et al. 2014; Liu et al. 2018). The identity of *H. hellenica* and *H. krugeri* emerged from a phylogenetic analysis of DNA sequence data for three gene regions (ITS, BT1 and TEF-1α), as well as their distinct morphological characteristics. Mating studies showed that *Huntiella hellenica* and *H. krugeri* were homothallic and heterothallic, respectively. All indications were that these two species are saprobes that grow on the freshly-exposed surfaces of trees.

The stump of *P. orientalis*, from which *H. hellenica* emerged, was sampled approximately two months after tree felling and it was also infected by the pathogen *Ceratocystis platani*, which causes a devastating disease in natural stands of *P. orientalis* in Greece. Colonszation of the stump with *H. hellenica* could have occurred on the freshly-cut surface with a contaminated tool as occurs for *C. platani* (Tsopelas et al. 2017) or was transferred by insect vectors.

The novel species described in this study showed typical characteristics of *Huntiella* spp. They grew rapidly in culture; their mycelium was white when young and turned dark with age. The one species that displayed a sexual state - *H. hellenica*, produced hat-shaped ascospores and had short conical spines on the ascomatal bases. Temperature is known to influence the ability of *Huntiella* spp. to produce a sexual state (Wilson et al. 2015) and this was also true for *H. hellenica*, which did not produce acomata below 25 °C.

Comparison of DNA sequence data for multiple gene regions is essential when seeking to identify species in *Huntiella* (Mbenoun et al. 2014; Liu et al. 2018). The three gene regions, selected for this purpose, have been used in previous studies showing that they can be collectively used to delineate species boundaries in the genus (Van Wyk et al. 2004, 2006, 2011; Kamgan Nkuekam et al. 2012; Mbenoun et al. 2014, 2016; De Errasti et al. 2015; Liu et al. 2018). However, analyses of individual gene regions revealed different levels of resolution, consistent with the results of previous studies on this group of fungi (Mbenoun et al. 2014; Liu et al. 2018). Thus protein coding genes, in this case BT1 and TEF-1α, provided the best resolution for species identification of *Huntiella*, while ITS sequences provided little information or no support.

Primers, developed to identify the mating type idiomorphs in *Huntiella* spp. (Wilson et al. 2015), were effective for this purpose in the present study. The results showed that *H. hellenica* has both mating-type idiomorphs and this explains the presence of sexual structures in all isolates derived from single hyphal tips. In contrast, the isolate of *H. krugeri* contained one mating gene and is clearly a heterothallic species of *Huntiella*, also consistent with the fact that the isolate produced only an asexual state. This result is also consistent with those of Liu et al. (2018) who showed that closely-related *Huntiella* spp. can have different mating strategies. Collectively, *Huntiella* spp. have a remarkable range of mating strategies, including homothallic and heterothallic species, as well as those exhibiting unisexuality (Wilson et al. 2015; Liu et al. 2018). The new species described here will contribute to future studies considering the evolution of mating in *Huntiella*.

The two new species of *Huntiella*, discovered in this study, bring the total number of species in the genus to 31. These are found in many different regions of the world and on a wide variety of woody substrates (Van Wyk et al. 2004, 2006, 2011; Kamgan Nkuekam et al. 2012; De Beer et al. 2014; Mbenoun et al. 2014, 2016; De Errasti et al. 2015; Liu et al. 2018). The renewed interest that these fungi have received during the course of the past decade has revealed unexpected complexity in their ecological interactions (Mbenoun et al. unpublished data), evolutionary history (Mbenoun et al. 2014; Liu et al. 2018) and reproductive biology (Wilson et al. 2015). The description of novel taxa, as reported in this study and the growing accessibility of whole genome sequencing (Wingfield et al. 2016), should enable new avenues of research that will contribute to a considerably better understanding of *Huntiella* species in the future.

## Supplementary Material

XML Treatment for Huntiella hellenica

XML Treatment for Huntiella krugeri

## Supplementary materials

Supplementary material 1 Figure S1. ML tree of *Huntiella* species generated from the ITS DNA sequence data Data type phylogenetic tree Explanation note Sequences generated from this study are printed in bold type. Bold branches indicate posterior probabilities values ≥ 0.9. Bootstrap values and posterior probabilities value are presented above branches as ML/MP/BI. Bootstrap value < 50% or probabilities values < 0.9 are marked with *. Nodes lacking the support value are marked with -. *Ceratocystis cercfabiensis* (CMW 43029) represents the outgroup. https://binary.pensoft.net/file/430555This dataset is made available under the Open Database License (http://opendatacommons.org/licenses/odbl/1.0/). The Open Database License (ODbL) is a license agreement intended to allow users to freely share, modify, and use this Dataset while maintaining this same freedom for others, provided that the original source and author(s) are credited.FeiFei Liu, Seonju Marincowitz, ShuaiFei Chen, Michael Mbenoun, Panaghiotis Tsopelas, Nikoleta Soulioti, Michael J. Wingfield

Supplementary material 2 Figure S2. ML tree of *Huntiella* species generated from the BT1 DNA sequence data Data type phylogenetic tree Explanation note Sequences generated from this study are printed in bold type. Bold branches indicate posterior probabilities values ≥ 0.9. Bootstrap values and posterior probabilities values are presented above branches as ML/MP/BI. Bootstrap value < 50% or probabilities values < 0.9 are marked with *. Nodes lacking the support value are marked with -. *Ceratocystis cercfabiensis* (CMW 43029) represents the outgroup. https://binary.pensoft.net/file/430556This dataset is made available under the Open Database License (http://opendatacommons.org/licenses/odbl/1.0/). The Open Database License (ODbL) is a license agreement intended to allow users to freely share, modify, and use this Dataset while maintaining this same freedom for others, provided that the original source and author(s) are credited.FeiFei Liu, Seonju Marincowitz, ShuaiFei Chen, Michael Mbenoun, Panaghiotis Tsopelas, Nikoleta Soulioti, Michael J. Wingfield

Supplementary material 3 Figure S3. ML tree of *Huntiella* species generated from the TEF-1α DNA sequence data Data type phylogenetic tree Explanation note Sequences generated from this study are printed in bold type. Bold branches indicate posterior probabilities values ≥ 0.9. Bootstrap values and posterior probabilities values are presented above branches as ML/MP/BI. Bootstrap value < 50% or probabilities values < 0.9 are marked with *. Nodes lacking the support value are marked with -. *Ceratocystis cercfabiensis* (CMW 43029) represents the outgroup. https://binary.pensoft.net/file/430557This dataset is made available under the Open Database License (http://opendatacommons.org/licenses/odbl/1.0/). The Open Database License (ODbL) is a license agreement intended to allow users to freely share, modify, and use this Dataset while maintaining this same freedom for others, provided that the original source and author(s) are credited.FeiFei Liu, Seonju Marincowitz, ShuaiFei Chen, Michael Mbenoun, Panaghiotis Tsopelas, Nikoleta Soulioti, Michael J. Wingfield
