# Supplementary figures and images for: Novel species of Huntiella from naturally-occurring forest trees in Greece and South Africa
Source: MycoKeys. 2020 Jul 10;69:33–52. doi: 10.3897/mycokeys.69.53205 (PMC7367892; doi:10.3897/mycokeys.69.53205)

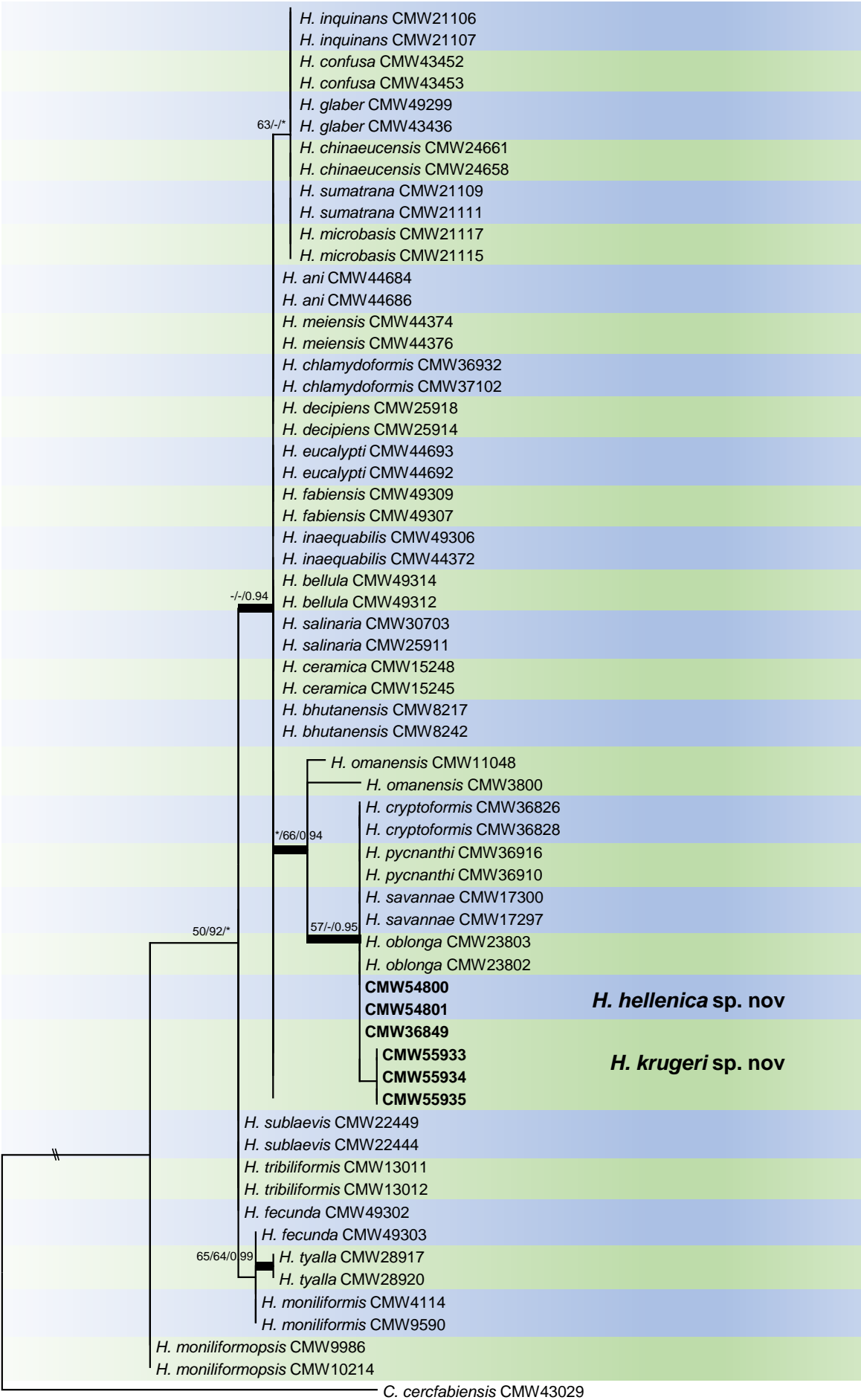

Supplement: Supplementary material 1 — Figure S1. ML tree of Huntiella species generated from the ITS DNA sequence data [file mycokeys-69-033-s001.pdf]

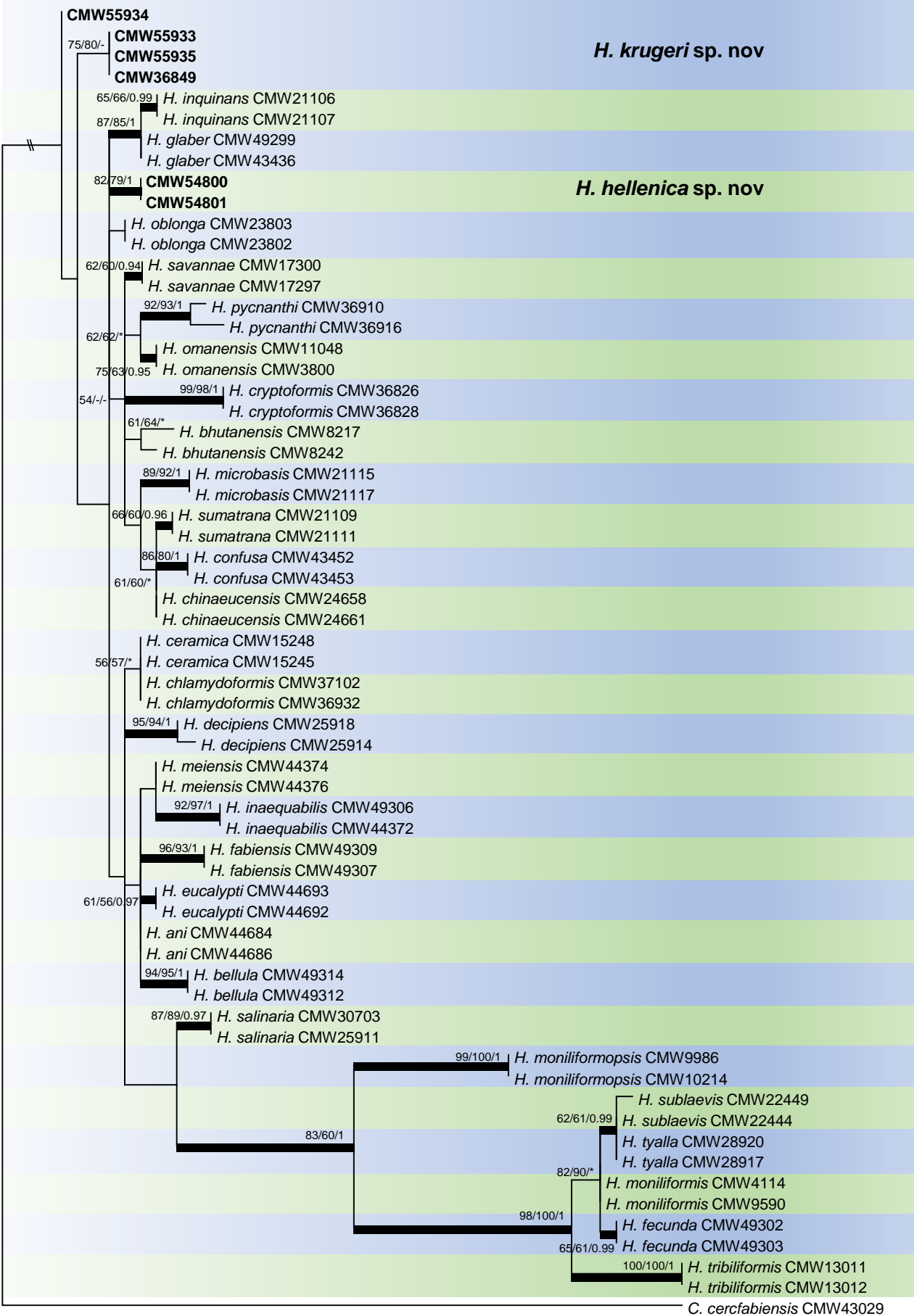

Supplement: Supplementary material 2 — Figure S2. ML tree of Huntiella species generated from the BT1 DNA sequence data [file mycokeys-69-033-s002.pdf]

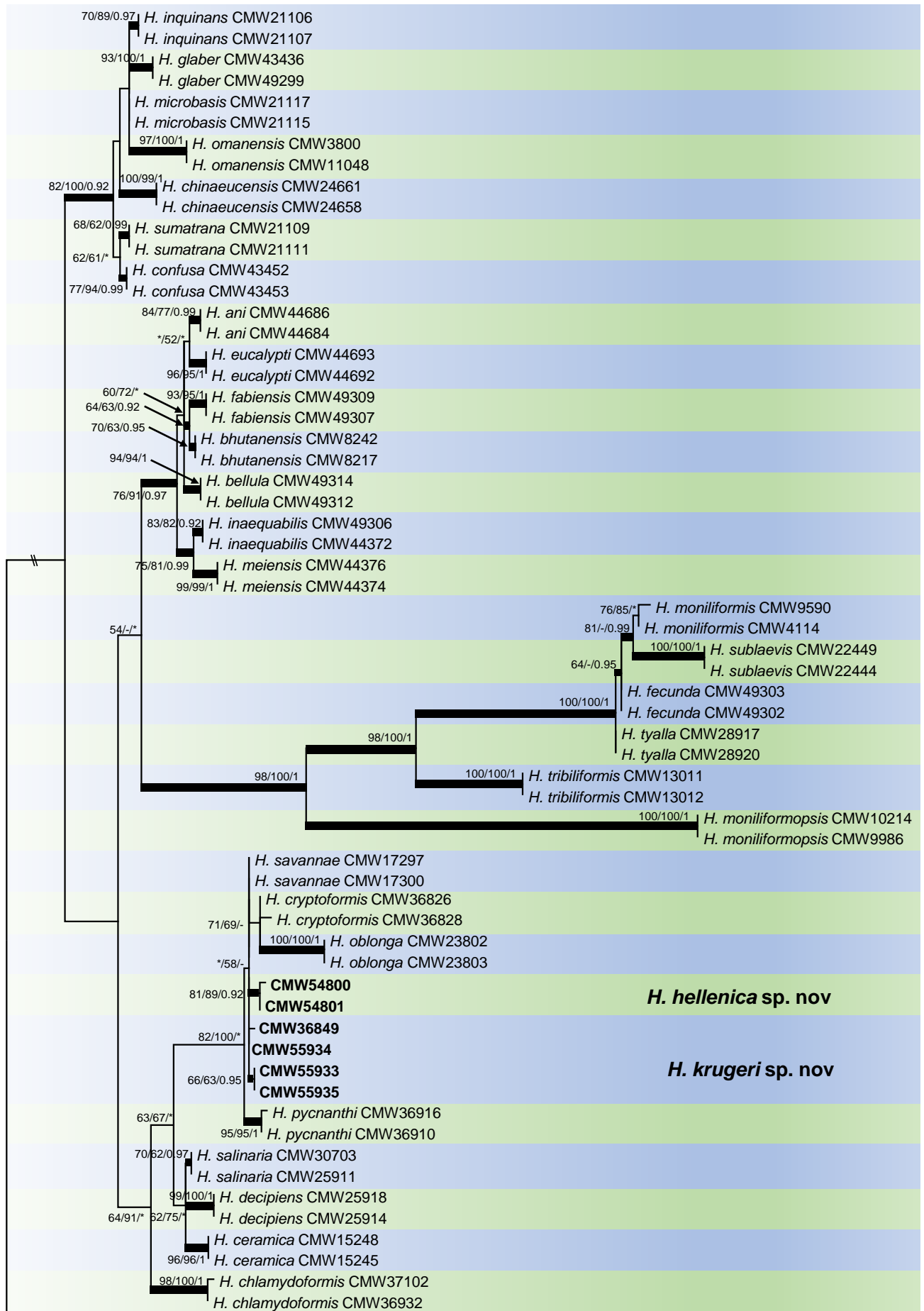

Supplement: Supplementary material 3 — Figure S3. ML tree of Huntiella species generated from the TEF-1α DNA sequence data [file mycokeys-69-033-s003.pdf]
